# What is Important to Older People with Multimorbidity and Their Caregivers? Identifying Attributes of Person Centered Care from the User Perspective

**DOI:** 10.5334/ijic.4655

**Published:** 2019-07-23

**Authors:** Kerry Kuluski, Allie Peckham, Ashlinder Gill, Dominique Gagnon, Cecilia Wong-Cornall, Ann McKillop, John Parsons, Nicolette Sheridan

**Affiliations:** 1 Institute for Better Health, Trillium Health Partners and University of Toronto, CA; 2 Dalla Lana School of Public Health, University of Toronto, CA; 3 Université du Québec en Abitibi Témiscamingue, CA; 4 University of Auckland, NZ; 5 Massey University, NZ

**Keywords:** multimorbidity, primary health care, patient experience, caregiver, Canada, New Zealand, qualitative, person centered care

## Abstract

**Introduction:**

Health systems are striving to design and deliver care that is ‘person centred’—aligned with the needs and preferences of those receiving it; however, it is unclear what older people and their caregivers value in their care. This paper captures attributes of care that are important to older people and their caregivers.

**Methods:**

This qualitative descriptive study entailed 1–1 interviews with older adults with multimorbidity receiving community based primary health care in Canada and New Zealand and caregivers. Data were analyzed to identify core attributes of care, important to participants.

**Findings:**

Feeling heard, appreciated and comfortable; having someone to count on; easily accessing health and social care; knowing how to manage health and what to expect; feeling safe; and being independent were valued. Each attribute had several characteristics including: being treated like a friend; having contact information of a responsive provider; being accompanied to medical and social activities; being given clear treatment options including what to expect; having homes adapted to support limitations and having the opportunity to participate in meaningful hobbies.

**Conclusions:**

Attributes of good care extend beyond disease management. While our findings include activities that characterize these attributes, further research on implementation barriers and facilitators is required.

## Introduction

Globally, health care systems are primarily oriented to deliver episodic care for acute conditions and are poorly calibrated to the needs of patients, particularly those with multiple concurrent chronic conditions (i.e., multimorbidity). People with multimorbidity report feeling overwhelmed managing their illnesses [1], experience a lack of care continuity and poor communication with care providers [2]. Care providers report frustration due to a lack of time in busy clinical environments, the absence of integrated electronic health records across care settings and a lack of applicable clinical practice guidelines to manage the multiple health and social needs that people present with [3,4]. Understanding what matters most to people in their care and creating the conditions to enable these needs to be met is required. However, such an approach requires a paradigmatic shift away from current health care systems which are rooted in a medical model where providers know best and patients are passive participants in their care.

Over the last several decades, with the rise of chronic conditions, a new wave of health care has been proposed and articulated in various models and frameworks including Wagner’s Chronic Care Model [5,6], Barr’s Expanded Chronic Care Model [7] and the various conceptions of *person and family centred care* [8,9,10,11]. These models and frameworks outline that it is appropriate that the balance of power between users and providers be equalized; that the patient be recognized as more than the sum and severity of their health conditions; and that patients have a right to participate in their care by providing input into care plans and articulating care goals [12]. While several empirical works and white papers endorse these goals of person centred care [13,14], the movement is stymied by care contexts that are structured and incentivized to support short clinical interactions and disease focused care.

Given the mismatch between the goals of person centred care and the disease focused orientation of current health care systems, it is not surprising that care experiences, particularly among people with multimorbidity, are generally reported as poor [15,16,17,18,19,20,21,22].

To improve these care experiences we must endeavor to understand what matters most to people. A systematic review conducted in 2016 by Mangin et al. [23] noted few tools that capture priorities and preferences among people with multimorbidity. Furthermore, existing definitions, conceptual models, and measurement tools on patient experience and person centred care are not always informed by patient or caregiver input [24,25,26].

In this paper we sought to explore what characterizes a good care experience and what is occurring in the patient’s and caregiver’s environment to enable this experience. If person centred care is truly a central aim of modern health systems, then some action is required to elicit, directly from patients and caregivers, what is important to them. Understanding attributes of a good care experience requires closer examination and can be used to guide efforts to implement models of care that truly reflect the needs of people and their caregivers and enable more positive care experiences.

This paper has two objectives: First, to capture what matters to older people with complex care needs and their caregivers (i.e., attributes of good care) and second, to outline the characteristics of these attributes (the core activities and actions that coincide with each attribute).

## Methods

This is a qualitative descriptive study entailing one-to-one semi-structured interviews with 172 patients and caregivers from 9 community based primary health care (CBPHC) sites located in Ontario, Quebec and New Zealand (NZ). A description of the nine case-study sites [27] and the process used to select them are described elsewhere [28].

A convenience sampling technique was used to recruit patients and caregivers. Patients were eligible for inclusion in the study if they were 65 years of age or older and had two or more chronic conditions. For NZ Māori, patient-participants were 50 years of age or older because, compared to the total NZ population, Māori experience chronic conditions earlier, more severely, and have a higher burden of multimorbidity. All participants were cognitively capable of participating in an interview. Non-English speaking participants were interviewed with the aid of an interpreter. Caregivers were eligible to participate if they were currently, or in the past, provided support for a patient that was enrolled in one of the nine case study sites. Administrative and front line staff from each of the CBPHC sites approached patients who met the inclusion criteria (in person or by phone) to assess interest and seek permission to have a researcher contact them. Among patients and caregivers who consented to participate, an interview was scheduled at a location of the participant’s choice (typically their home or the main site of care/primary care clinic). Ethics approval was given by the University of Auckland Human Participants Ethics Committee, University of Toronto Research Ethics Board, Michael Garron Hospital Research Ethics Board and Bridgepoint-West Park Toronto Central CCAC-Toronto Grace Research Ethics Board. Interviews were audio-recorded and transcribed verbatim by a transcriptionist. Interviews were not returned to participants for comment. Table 1 outlines the characteristics of patients and caregivers across all study jurisdictions.

**Table 1:** Participant Characteristics.

Clients	Toronto	Quebec	New Zealand	Across all 3 Jurisdictions

	n = 32	n = 12	n = 39	n = 83

**Translated**	24%	0	0	10%
**Age**				
*<50*	6%	0	0	2%
*50–64*	13%	0	36%	22%
*65–74*	25%	50%	23%	28%
*>75*	56%	50%	41%	48%
**Sex**				
*Female*	91%	50%	62%	71%
**Ethnicity**	44% East Asian 25% European 19% Caribbean	92% French Canadian Caucasian 8% South Asian	62% Māori 36% NZ European 3% Other European	29% Māori 18% Canadian Caucasian 17% East Asian 17% NZ European 11% European 7% Caribbean 1% South Asian
**Living Arrangements**				
*Lives alone*	44%	83%	31%	43%
*Lives with at least 1 other person*	56%	17%	69%	57%
**Number of Conditions**				
*Coronary Obstructive Pulmonary Disease*	9%	17%	18%	14%
*Cancer in the last 5 years*	16%	17%	5%	11%
*Asthma*	13%	17%	23%	18%
*High blood pressure*	63%	87%	36%	51%
*Ischemic Heart Disease*	16%	0	46%	28%
*Diabetes*	41%	33%	62%	49%
*High Cholesterol*	66%	33%	8%	34%
*Stroke*	22%	75%	13%	25%
*Arthritis*	75%	58%	51%	61%
*Other (Dementia, Alzheimer’s, Cataracts, Hearing Impairment)*	53%	25%	33%	40%
*Chronic Pain*	78%	42%	46%	58%
*Mental Health (Anxiety, Depression)*	50%	17%	33%	37%
**Caregivers**	39	9	41	89

**Translated**	23%	0	7.3%	13%
**Age**				
*<50*	18%	0	20%	17%
*50–64*	36%	67%	48.8%	45%
*65–74*	27%	11%	17.1%	20%
*>75*	21%	22%	14.6%	18%
**Sex**				
*Female*	69%	89%	63.4%	69%
**Ethnicity**	49% East Asian 21% European 13% Canadian, Caucasian 10% Caribbean 8% South Asian	78% French Canadian Caucasian 22% European	46.3% Māori 34.2% NZ European 17.1% East Asian 2.4% NZ European and Māori	29% East Asian 21% Māori 16% NZ European 13% Canadian Caucasian 11% European 4% Caribbean 3% South Asian 1% NZ European and Māori
**Relationship to Client**				
*Child*	54%	56%	48.8%	53%
*Spouse*	36%	22%	43.9%	38%
*Other*	10%	11%	7%	9%
**Living Arrangements**				
With Client	74%	56%	73.2%	72%
Lives separately	26%	44%	27%	28%
**Chronic diseases managed by Caregiver**				
*Coronary Obstructive Pulmonary Disease*	8%	0	10%	8%
*Cancer in the last 5 years*	10%	22%	12%	12%
*Asthma*	13%	0	10%	10%
*High blood pressure*	64%	22%	54%	55%
*Ischemic Heart Disease*	41%	12.5%	32%	34%
*Diabetes*	59%	33%	46%	42%
*High Cholesterol*	49%	12.5%	29%	47%
*Stroke*	49%	44%	24%	37%
*Arthritis*	56%	12.5%	27%	38%
*Other (Dementia, Alzheimer’s, Cataracts, Hearing Impairment)*	62%	56%	7%	36%
*Chronic Pain*	54%	12.5%	15%	31%
*Mental Health (Anxiety, Depression)*	67%	12.5%	30%	44%
**Support Provided by Caregiver**				
*Personal care*	76%	56%	41%	57%
*Day-to-day assistance*	95%	56%	85%	85%
*Household chores*	97%	78%	83%	88%
*Additional support: Companionship, Decision making*	95%	67%	100%	93%

## Analysis

The research question: *“What is most important to patients and their caregivers in their care?”* was applied to the dataset. Consistent with a directed content analysis approach [29], specific codes (i.e., common passages of text) which the team felt would help them understand the attributes of care that were important to patients and caregivers were selected for review. These codes included perceptions of unmet need; feelings related to health, symptoms and limitations; perceptions of care provided to them; caregiver experiences and needs; self-management support; and roles and relationships with a range of providers and services including primary care, homecare; hospital; emergency department, etc.) These codes were analyzed in-depth using an inductive approach which entailed reading all text line by line and organizing similar text into core categories. Four members of the research team (KK, AP, AG and SD) conducted the analysis individually and then came together periodically to discuss emergent categories – each of which represented an attribute of good care from the perspectives of patients and caregivers.

Part way through the analysis the coding framework was presented to the broader team for feedback (including those who had conducted the patient and caregiver interviews in other jurisdictions). It was agreed that the selected codes were appropriate and the emergent categories consistent with expectations of what was relevant to patients and caregivers.

The data were recoded using the categories as an organizing framework to ensure all relevant content was captured. Any other content that was thought to be relevant were also selected and categorized separately for further discussion. Modifications to the categories were made by three members of the team (KK, AP and AG) following several in-depth discussions (some of the categories were merged and renamed). A descriptive memo was written for each attribute detailing the key characteristics.

## Findings

**Figure 1:**
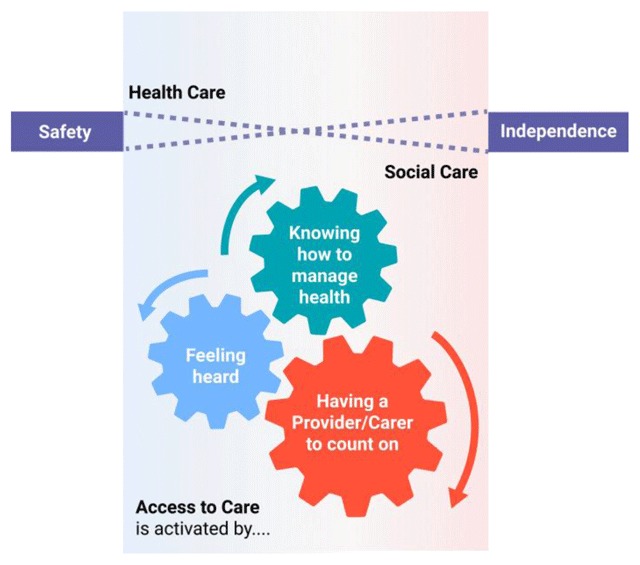
Intersection of Attributes.

Six core attributes were identified. Each of these attributes were relevant to all study jurisdictions and represented within both patient and caregiver groups: feeling heard, appreciated and comfortable; having someone to count on; easily accessing health and social care; knowing how to manage health and what to expect; feeling safe; and being independent. A description of each attribute along with example quotes from a range of cases and jurisdictions is provided below. Table 2 (at the end of the findings section) provides a summary of each attribute and corresponding characteristics. During the analysis it was clear that each attribute was not mutually exclusive, but rather complementary and informative of other attributes. As a result, the attributes were organized into a schematic (see Figure 1 at the end of the Findings section).

**Table 2:** Provider Attributes and Supporting Activities.

Attribute	Example Characteristics

Feeling Heard, Appreciated and Comfortable	Talk to patient and caregiver like a friend indicative through tone of voice, facial expressions and probing follow-up questions provider is humble, uses humor, and is more relaxed
	Patient/caregiver and provider put themselves in the shoes of the other and attempts to understand the others constraints willing to sacrifice/compromise, tolerant of the other’s experience and perspective
	Focus on the person outside the diagnosis probe for personal context outside of health care needs, to understand family/social life, interests, and priorities
	Take time with the patient and family patient and family do not feel rushed during interaction provider is present and listens intently
	Consistent people provide care to increase patient and caregiver comfort
	Patients’ providers talk to one other, sharing appropriate information so everyone knows what is going on
	Provider, patient and caregiver speak the same language or have appropriate translation available
	Caregiver’s experience is acknowledged identify them and explore resources to manage burnout include them in decision making

Having Someone to Count On	Having a trusted ‘go-to’- person (typically a paid provider) who is: responsive and can connect to the broader team when needs arise accessible to the patient/caregiver (direct contact details provided)
	The counted on person responds quickly or manages expectations about response time and: keeps track of patient appointments provides reminder calls (re: appointments and follow-up) conducts or arranges home visits works with patients and caregivers to address problems as they arise to avoid isolation and unnecessary emergency visits goes the extra mile (e.g., drives patient to an appointment so the caregiver can have a break; picks up and drops off medications; arranges translators; ensures that transportation services align with appointment schedules, etc.)

Easily Accessing Health and Social Care	Access enabled by having a ‘go-to’ person who can connect and facilitate access to health and social resources (as outlined above)
	Providers span boundaries/wear multiple hats so both health and social needs can be met simultaneously (such as providing fresh food in primary care clinics or liaising with housing supports)
	Ensure services are *useful* and practical (such as having food delivery *with* instructions on how to prepare the food or having assistive devices delivered *and* installed).
	Offer different methods of service provision in clinic, home visits, videoconferencing proactive approach to service offerings same-day visits for urgent needs, emergency response programs
	Health and social care resources offered under one roof or in close proximity coordinate services between health and social care sectors and agencies

Knowing How to Manage Health and What to Expect	Use lay language (avoid complex medical terms)
	Provide clear explanations as to *why* certain treatment options are recommended and what to expect
	Instill confidence in patients and caregivers in self-management provide instructions, written list of steps, “how-to” guides on symptom management increase time, follow-up and discussion during appointments be mindful of their readiness for change when recommending treatment/suggestions
	Accept a ‘trial and error’ approach to health management try different treatment/medication regimens, work closely with team (including the patient and caregiver) to modify plan, check-in continuously explain why certain things may not be possible, and propose alternatives
	Involve caregivers in discussions and work together to implement a plan to manage health and social experiences
	Plan ahead have conversations about current capacity, long-term supports needed and as well as end of life preferences
	Work with patients and caregivers to come to terms with current health by modifying activities or ceasing activities (such as driving) if unsafe
	Tell patients and caregivers what services they are eligible for

Feeling Safe	Provide patients and caregivers access to needed mobility aids and offer training in their use inside and outside the home
	Ensure patients have access to personal resources (e.g., finances, caregiver support) to obtain needed equipment/mobility aides
	Ensure that the caregiver is able to safely do transfers and personal care without putting health at risk
	Work with caregivers in their home to address complex care needs of patients (such as behaviors and unpredictable events that typically arise with dementia)
	Provide caregivers with additional supports to offer *peace of mind* so they are able to attend appointments or social outings and know the care recipient is safe caregivers must be able to trust and easily access these resources – (e.g., access to consistent providers who understand the needs of the patient).

Being Independent	Explore opportunities for patients and caregiver to participate in enjoyed activities (connect with friends, partake in hobbies, travel)
	Ensure caregivers are able to have a “true” break for respite homecare hours may have to be adjusted as duration of homecare/day programs/respite care is often not long enough for caregiver activities (e.g., errands, employment, vacation etc.)
	Give choices in a care plan (if desired by patients and caregivers) so they still feel in control
	Explore how the patients built environment can be conducive to enabling autonomy (assisted living/supportive housing options to support help with instrumental tasks)

### Feeling Heard, Appreciated and Comfortable

Patients and caregivers talked about the importance of feeling *human* during interactions with care providers. When patients and caregivers felt a strong connection to their care providers they could “*relax with them.”* They stated that *“it’s not so clinical”* and *“they are like an extended family member.”* The relational aspects of care were characterized by providers being present (listening intently), asking probing questions beyond illness and physical symptoms, taking the time during visits and using a gentle demeanor during care interactions. This enabled patients and caregivers to relax, open up and discuss what was important to them.

*“First of all, he treated me as an equal. He listened to me. He also asked questions and asked the right questions. [Other doctor] did not generally ask questions. I have to tell him everything and ask is this possibly due to this? What do you think of this? In other words, I have to take the initiative and even opening the subjects and things pertaining to my care.* (Canada, Case 3, Patient 12)

Patients felt heard when providers respected their opinion and preferences:

*“She [the provider] gives me good advice. She keeps me informed, she doesn’t insist on anything. I am the one who decides. If I am not feeling good about something, she helps me find something else.”* (Canada, Case 4, Patient 2)

The caregiver here appreciated not being looked down upon, having ample time with care providers and responsive service provision:

*“I just feel they don’t look down on us. They don’t treat us like we’re an idiot. Because, with my husband’s illness, especially, because a lot happens and it’s a very slow thing, and…they spend as much time as need[ed] with you.” Yeah. And, I’ve also found they are very onto it- as soon as they think something is wrong, they don’t just say, “See how you go, come back next week.” They get straight into it.”* (NZ, Case 3, Caregiver 4)

Shared ethnic and cultural backgrounds among patients, caregivers and providers fostered trust, connectedness and understanding:

*“I feel that they give a different kind of care, it’s…for Mum, who is having strangers coming into the house when I’m not home, having a Māori walk in and know that, you know, you take off your shoes and go make your cup of tea and bring it to the room, and, you know…is kind of really important.”* (NZ, Case 3, Caregiver 12)

Some patients and caregivers recognized the time constraints of health providers and appreciated their time with them, even if limited.

*“They are just so energetic, and they’re passionate. They just commit themselves, they commit themselves to me while they’re here, and I know very well they have many other clients. So I try and minimize my time here for them, but before they get here, I highlight all the areas that I want to cover. Before they leave, I just put, ‘Let’s revise these areas next time we meet”* (NZ, Case 2, Patient 24)

### Having Someone to Count On

It was important for both patients and caregivers to have someone they could rely on in times of need. This person was often a paid care provider. This ‘go-to’ person was most helpful when they could access other team members (paid professionals) and resources needed by the patient or caregiver; when they could be reached easily (by phone or text message) and respond relatively quickly or manage expectations about response times. By keeping track of appointments and treatments, access to care was generally more convenient. This person served as the conduit to a broader array of people and resources, including social supports and activities, not just health care services.

Having quick access to reliable care providers gave caregivers peace of mind:

*“…with having the nurse come, it just gives us peace of mind, and I know I can, where it’s very difficult to try and contact a doctor, or you can through a nurse at the medical centre, but [name removed], she encourages us, if we need, just to ring her.”* (NZ, Case 2, Caregiver 17)

Furthermore, quick access to a reliable provider who was familiar with the patient and caregiver could mitigate a health crisis:

*“We had one just the other day, because he was having a hard time breathing, and we called and, uh, the nurse answered right away. Uh, she got someone to come down and see him… she came down to see him actually, and she called the doctor here immediately, and the doctor sent a prescription and all…the she knew all about him, send a prescription here, he’s taking them now…”* (Canada, Case 2, Caregiver 2)

Another caregiver appreciated help planning care across time and providers:

*“Yeah, they’ll write things down. And they also ring up to remind me of my appointments, which I forget otherwise, they’re beautiful. They’re awesome.”* (NZ, Case 2, Caregiver 25)

Patients and caregivers appreciated having someone that was both accessible and able to coordinate with other providers:

*“Yeah, absolutely. She’s only a phone call away and she has the inside running of the know…She can make appointments with the doctor.”* (NZ, Case 2, Patient 29)

### Easily Accessing Health and Social Care

Easily accessing health and social care was facilitated by the ‘go-to’ person as illustrated above. Importantly, it was not enough to *just access* services, the resources accessed had to bring *value* and *meaning* to the person. The value of service was enhanced by providers and organizations fulfilling more than one role (supporting health and social care needs simultaneously) such as a primary care clinic that had fresh food available or medical and financial supports available under the same roof.

*“We’re fine [referring to relationship with doctor]. Yeah, I love coming here. I can eat. I just come and I get food and all these things.”* (Canada, Case 3, Patient 9)

Patients ‘symptoms were unpredictable and fluctuated from one day to the next making it difficult to schedule and attend appointments outside the home. A patient talked about the importance of flexibility given the unpredictable nature of illness:

*“I can never tell how my physical condition is going to be from day to day, so I frequently have to cancel and reschedule my [appointments] at the last minute. I know it’s also trying to tell people there’s a charge if you don’t- if you cancel late. What am I supposed to do? I do not know until the day when I’m supposed to be traveling whether I’m in the physical condition that I’ll be able to travel.”* (Canada, Case 3, Patient 10)

Having access to care while at home, from a visiting provider or a ‘virtual visit’ was appreciated by homebound chronically ill patients and their caregivers. A caregiver from Ontario shared her experience with such a program:

*“Just amazing, so we had…They had a panel of like 12 people from the dietician to the physiotherapist to the neurologist […] I don’t remember who they were and we were on Skype with them and we were basically allowed to speak with them for about almost 14 minutes.”* (Canada, Case 2, Caregiver 6)

Patients valued having quick access to care when experiencing a health issue. When feeling unwell, this patient was able to hit a call bell [a built in feature in her apartment building] and get an immediate response:

*“I’ve got a button right down there. And it’s for 911 [emergency] and [name of organization]. And I pushed the [button] …I still don’t know what happened. But I was dizzy and everything. But the girls were so good.”* (Canada, Case 1, Patient 11)

Patients and caregivers appreciated when providers drew other helpful resources to their attention:

*“She definitely helps me, things that I am entitled to that I would never had known about and she’s brought them to my attention, she’s taken me to the [housing organization] to help me with things, she’s always asking me if there’s any way she can help me and…They are a wonderful combination, they really are. Super Girl and Super Woman.”* (NZ, Case 2, Patient 31)

Similarly, in the excerpt below a volunteer connected a patient and their caregiver to financial benefits they did not know about. Prior to that they were spending their limited resources unnecessarily.

*“So I made the arrangements, get all the doctor’s letters and everything, make an appointment, accompany them for the interview. So eventually they got their disability social benefit. So from then on, their drugs are free.”* (Canada, Case 1, Caregiver 9)

On the other hand, some services had limiting characteristics that did not fully meet the patients and families’ needs. In other words, the resource was provided but was not fully accessible to patients:

*“…the occupational therapy rang me on the Tuesday that was, I think, the bed arrived a week before, I think, and she said, ‘oh, has the bed arrived?, and I said yes and explained that it was in the garage, and, you now. And she was really annoyed with the company who was supposed to pull it out. So I think there could be a bit more communication.”* (NZ, Case 1, Patient 7)

Similarly, this patient talked about the limitations of a food delivery program:

*… “I didn’t know how to prepare them [raw food]. If they had sent along paper saying this is a recipe for how to prepare the stuff, it would have been different, but they just send it out expecting: Look we have this great, big box of fruits and vegetables! And you’re staring at them going, well I can eat these five but these 20 goes in the garbage because you don’t know what it is.”* (Canada, Case 3, Patient 1)

### Knowing How to Manage Health and What to Expect

Knowing how to manage health at the present time and into the future was contingent on having clear explanations from health care providers- who spoke in lay language, avoided “big flash words,” and used communication aids like diagrams to illustrate key points. Clear explanations were often coupled with taking time with the patient and family to verify understanding, provide opportunities to collaborate and negotiate next steps.

*“She’s [health provider] a very relaxed type of person. She’s not, you know, in your face or anything like that, she just sits back and, you know, explains things to me and talks about what problems I’ve got, you know. And why they’re putting me on this or why they’re putting me on that.”* (NZ, Case 1, Patient 7)

In addition, knowing why certain treatment options were being recommended by the doctor, nurse practitioner or nurse was important and often linked to adherence:

*“And the medication they’ve given me, they’ve explained to me what it is for. And because I understand what it is for now, I take them. Whereas before, oh no, didn’t know, so out the window it went.”* (NZ, Case 3, Patient 1)

Managing illness (as is often the case for people with multiple chronic health problems) was unpredictable and required some level of trial and error or continuous adaptations with the care provider.

*“After you have taken the medication for a day or two, he would phone to ask if you have any reactions or the outcomes, like how is the result. Through this I think he is very caring.”* (NZ, Case 1, Caregiver 38)

For patients who had dementia, caregivers tried different approaches (such as purchasing gates with locks to prevent wandering, and installing alarms in the home) to keep their family members safe. A caregiver appreciated having a care provider come to her home to teach her how to manage her mother’s unpredictable behaviors:

*“She [social worker] contacted the different departments about the dementia behavior, and asked them to come to me to interview and teach me how to handle his behavior.”* (Canada, Case 1, Caregiver 3)

Having the ‘tools’ to manage health was critically important and included having a list of results to take from provider to provider (a traveling record) and having someone accompany the patient to a doctor’s appointment (to either drive, take notes, interpret or advocate if needed).

*Yeah. Because [care provider] prints it out for me, my blood pressure results and that, she’ll print it out. And that’s, like she said, that’s just in case I might have to go and see [other care provider] sometime. And I can take the list with me, so it’s in a folder.* (NZ, Case 3, Caregiver 16)

Caregivers played a huge role in helping patients figure out how to manage illness; stay on top of things; liaise with the care team; decode language that was hard to understand; get explanations from care staff and organize follow-up appointments to manage health.

It was also important to ‘future proof’, or get things in order for the future. This included getting onto a long-term care wait list (particularly important for caregivers with capacity limitations) and end-of –life planning.

*“So I’m thinking for the future I can put him on a wait list [for long-term care]. It gives me time to shop around, look for the best place…”* (Canada, Case 2, Caregiver 13)

### Feeling Safe

Safety was a major concern among caregivers. Caregivers had to carefully navigate the balance between *restricting* behaviors and actions of those they cared for with enabling or honoring the patients’ preference to do activities independently.

This caregiver had to constantly monitor her mother to ensure she could do activities in a safe way:

*“If she’s around [patient], constantly have to make sure that she’s okay. If she’s walking downstairs, okay, on the main floor, I make sure that she’s okay. I turn on the TV on so that she can watch the program. Or I get the iPhone so that she can play something. I get her occupied. Or if she likes to go outside, I want to make sure that she’s okay. I watch her in the vegetable garden picking the stuff, you know, so she’s safe. So if she’s around then I have to make sure that her meals- breakfast, lunch and then her afternoon tea, and that sort of thing. So my day is gone really fast.”* (Canada, Case 1, Caregiver 26)

A caregiver described the dangerous events that prompted her to move her mother into a residential care facility. There were signs that her mother’s dementia had reached a point where she needed 24/7 care and the caregiver was worried about her mother’s safety.

*“She left the stove on. You know and fat all on the stove and stuff like that. And when I asked her about it, she would, she was angry, she didn’t want to talk to me. She wanted me to leave. She didn’t wanted anything to do with me. Because she knew that she was not coping.”* (NZ, Case 3, Caregiver 10)

Some caregiver’s resisted recommendations made by care staff if they felt it could potentially lead to an unsafe situation:

*“I wasn’t the type of person that was assertive; they would have sent her home. And she would have had an accident, and she would have killed herself. I am absolutely certain of that. I mean I have to physically take her car away from her because even though she knew she wasn’t allowed to drive…”* (NZ, Case 3, Caregiver 10)

Patients expressed their own personal fears including slipping in the shower or falling and getting lost outside:

*…I don’t have much confidence because I’m all alone. And the if I walk outside, if there’s some kind of trouble, it might cause a problem…. I may fall down”* (Canada, Case 1, Patient 16)

A patient aptly stated:

*“It’s clear that having someone to assist me to take a shower helps me not to be afraid.”* (Canada, Case 5, Patient 2)

Some caregivers were dealing with their own health issues which impacted their capacity to help:

*“Well, he’s afraid to walk out because he’s going to…If he fall…He’s still a big guy. And if he falls here….I mean he walks with a walker in the apartment. And years ago he never did. You know, he’d have a cane or he would hold onto the walls. You know, he’s afraid. I [caregiver] won’t be able to pick him up.”* (Canada, Case 3, Caregiver 5)

Patients and caregivers wanted to adapt their homes (with grab bars, appropriately sized wheel-chairs and walkers) to support confidence and ease of mobility both inside and outside the home.

*“I would love to have a shower but at the moment I can’t step on onto the bath. But they’re going to take all that out for me […] so I can just walk into the shower.”* (NZ, Case 1, Patient 6)

### Being Independent

The health care system, oriented toward safety sometimes restricted patient independence. The patient detailed below was living with her children and did not want to be put in a nursing home. She preferred to age in her own home.

*“She [daughter] wants to put me in a home. I said no, I’ve got a home and I got my home by myself with [housing provider]. I had a house in [city] then I had to transfer.”* (NZ, Caregiver 19, Case 2)

Caregivers appreciated when providers enabled them to get out and do activities that *they* enjoyed:

*“Well if it wasn’t for [providers], you know, I would never have been able to go out fishing. That’s, that’s for starters. I went out 5 times last year, during the summer months, and they were so rapt that I did get out.”* (NZ, Case 2, Caregiver 18)

The act of caregiving limited caregivers’ independence. It was difficult for caregivers to make plans too far from home, go on vacation and partake in social activities. Some caregivers constantly checked in with their loved ones if they were concerned about leaving them alone for too long. For some, this stemmed from a lack of trust in care providers in their absence, (particularly if staff turnover was high). Patients with dementia were uncomfortable with new staff which limited the caregiver’s ability to get away and have a break.

*“Because of her, I change everything from before. Such as my eating, my working hours, my life, my living habits, including my eating.”* (Canada, Case 1, Caregiver 14)

Caregivers had to negotiate service hours (taking them in longer blocks) to allow them enough time to get longer breaks or get other errands done:

*“…I take it as a block. Like they come 3 days. Because what’s the point of coming one hour in the morning, one hour at lunch? I can’t do anything. I have to be here all the time. So the only thing I said, okay, you come for 4 or 5 hours. At least I can go for a doctor’s appointment, I can go for eye check-up or shopping, grocery shopping, thing kind of thing.”* (Caregiver, Case 2, Caregiver 11)

On the other hand, the health care system also enhanced *independence*. Paid supports such as respite care (if structured in long enough blocks of time), adult day programs where patients could spend time with their peers, allowed both patients and caregivers to feel a sense of independence and contentment. Even small things like providers’ arranging to drive and accompany a patient to an appointment, even when a caregiver was present, freed up time for caregivers to get other activities done or have a much needed break.

*“It’s like when Mum has to go to the hospital and stuff like that, appointments, they come pick her up and take her there, bring her home. It saves me doing it.”* (NZ, Case 2, Caregiver 19)

Finally, supportive housing in the Canadian context provided a structure that enabled patients to easily self-manage and call on providers, if and when they needed supports (such as taking food out of the oven) or hitting a call bell if they felt dizzy.

*“Like I can’t use my oven and stuff like that because I’m shaky. So that’s limited. The girls [personal support workers] come in and help me. If I put something in the oven, I have to get them to come and take it out.”* (Canada, Case 1, Patient 11)

## Discussion

The paper provides an overview of what matters most to older adults with chronic conditions and the people who care for them. The paper draws from 172 in-depth interviews with patients with complex care needs and their caregivers from a range of jurisdictions in two countries. Our sample was ethnically diverse including participants who identified as Māori, New Zealand European, East Asian, European, Caribbean and South Asian; 10% of participants were non-English speaking. Core attributes of care that were important to patients and caregivers were identified—feeling heard, appreciated and comfortable; having someone to count on; easily accessing health and social care; knowing how to manage health now and in the future; feeling safe; and being independent. We explore our findings further under three broad categories: the social side of care, ‘managing’ health and trading off.

## The Social Side of Care

Each of the attributes, articulated by patients and caregivers pointed to the importance of the social side of care, including the importance of relationships between patients, caregivers and the care team. Access to social and instrumental supports was critical and this ranged from healthy food, to reliable transportation, and access to hobbies of interest.

The human side or relational aspects of care are often taken for granted in favor of foci on cure, treatment and symptom control. Busy health care environments afford little time for providers to develop relationships with patients and families, to reflect, listen and engage meaningfully. It is during these encounters that trust can be established, comfort increased and priorities and preferences of all parties revealed—critical to creating a care plan that works. This stands in stark contrast to medical models of care and clinical practice guidelines that create parameters around the roles and activities of care teams and perpetuates an orientation toward disease management.

In some cases, patients and caregivers recognized the constraints of the provider and adjusted their expectations and actions accordingly, choosing priorities to discuss now versus later. In these cases, they seemed cognizant of the providers’ constraints within the busy health setting. Patients in these cases did not express dissatisfaction with their care; they simply acknowledged the reality of their situation. This type of situational awareness may have drawbacks as well as benefits. For example, patients may lower their expectations to a point where their needs are not fully met because they censor the conversation with providers, potentially leaving out important aspects of their needs and preferences.

There is some emphasis in the patient centered care literature for providers to put themselves in the shoes of the patient it is also incumbent upon patients to put themselves in the shoes of the provider. Like any relationship, putting yourself in the shoes of the other, can foster empathy within a relationship and facilitate a strong therapeutic alliance. The concept of counter-transference is relevant here- the “sensitivity and insight into the reactions of both parties [9, p. 236].” The management of multiple complex health and social needs in a system of care that is not wholly integrated requires some level of sensitivity to the other party. Perhaps this should be considered an essential component of person centered care; a more realistic goal as we look to create conditions in health systems to foster better care experiences. However, caregivers may find themselves doing more than they should, or doing things they do not feel confident doing and being empathetic to providers will not solve this issue. The patient/caregiver and provider relationship is one of unequal power so there is a risk that patients and caregivers will limit their demands based on available resources.

## ‘Managing’ Health

Having a “connector”- someone to rely on in times of need was critically important to people managing complex health needs. In a paper by Haggerty entitled “Ordering the Chaos for patients with multmorbidity” [30] the importance of having a core coordinator to ‘connect the dots’ for people with multiple conditions was discussed. She suggests this role should be fulfilled by the person with the most comprehensive knowledge, pointing to the general practitioner. While this could be the case, our study also shows that such roles are effectively undertaken by nurse practitioners, nurses and community social workers (particularly in NZ) as well as unpaid family and friend caregivers or volunteers. When the core ‘go-to’ person was not the physician, it freed up the physician’s time to do other things. Care delivery appeared streamlined as as long as the primary care team still effectively communicated with the connector. The importance of care navigators (also referred to as case managers or care coordinators) are well cited in the literature [31,32,33] but coordinators often get stuck within a specific sector or boundary (e.g., such as hospital but requires the mobilization of resources in the homecare or housing sector). These findings relate to the literature on inter-organizational work and the importance of individuals working across boundaries or ‘boundary spanners’ [34,35,36] to meet the diverse needs of patients. In our study, ‘go to’ people had access to and relationships with providers in other sectors and were able to access resources from them. When providers could cross boundaries (i.e., organizations and sectors)—either by wearing multiple hats (e.g., providing both health and socially oriented care), or working in a space that allowed patients and caregivers to easily access social resources (fresh food, exercise programs, community gardens, opportunities to engage with peers) it facilitated an overall, better health care experience and easier access to needed resources that were meaningful.

*Knowing how to manage health and what to expect* was important for both patients and caregivers who at times found themselves with no clear answers as they attempted to manage multiple and often conflicting treatment regimens. There is a burgeoning literature on self-management support including tool-kits and taxonomy’s that are meant to operationalize self-management activities. For example, the ***P**romoting **R**esilience, **I**ndependence and **S**elf-**M**anagement **S**upport* (*PRISMS*) taxonomy outlines 14 components or ‘tools’ that support self-management and care of people with chronic conditions including information about conditions and available resources, an agreed upon clinical action plan, regular review, ongoing monitoring, practical support of medicines and behaviors, provision of equipment, easy access to advice and support when needed, learning how to communicate with providers, training for everyday activities including self-management of conditions, coping, access to social support and advice on how to handle lifestyle stressors [37]. As acknowledged by co-author (NS), in a previous paper [38], this taxonomy does not provide enough detail on the relationship and engagement between patients, caregivers and providers. Our work also acknowledges the ‘trial and error’ approach to managing multiple chronic conditions, and the ‘work’ required by both the provider and patient/caregiver to continually communicate, and make adaptations. The ‘work’ needs to be situated within a trusting relationship where patients and caregivers feel comfortable expressing their needs and disagreements. The continuous ‘back and forth’ and elicitations of preferences and goals supports the idea of ‘minimally disruptive medicine’ – improving outcomes that matter to people with the smallest burden of treatment possible [39].

## Trading Off

Our analysis suggests that not all attributes are perceived equally. In addition to overlapping and intersecting, the attributes may trade-off for patients and caregivers. The clearest example was the trade-off between ‘safety’ and ‘independence.’ While both attributes were important to patients and caregivers, for caregivers, patient safety was prioritized over patient independence. Achieving the right balance between safety and independence was difficult and caregivers often experienced guilt and uncertainty with the decisions and actions they took. Caregivers witnessed angst among those they cared for who yearned to do activities that placed them at risk. This trade-off was particularly apparent among people with dementia where caregivers were concerned about patient’s safety and well-being, would limit a patient’s outside activity and hobbies unless they were there to closely monitor them. A trade-off was mitigated when providers included caregivers in the care plan and were aware of their needs as well as the patient’s needs. For example, arranging time for respite care so the caregiver could participate in an enjoyed activity; scheduling longer blocks of care; and arranging transportation for the patient to get to and from appointments; took the burden off the caregiver. Another example is the personal modifications made by patients and caregivers themselves as they adjusted to their reality with illness. In these cases, changes to regular activities and routines were self-imposed but, at times, led to mental anguish and a restricted life (e.g., patients would stay indoors and experience sadness at not being able to do an enjoyed activity like playing sports or gardening). The more the care team could do to help patients and caregivers keep some semblance of a ‘normal’ life was appreciated (which could include access to day program, home and virtual visits with the care team and access to volunteers).

Finally, as noted in Figure 1, it seemed that certain attributes activated other attributes. Our research suggests that access to care is activated by having someone to count on (a key point person who is responsive). When the provider/team is responsive and open to patient and caregiver needs, they are more likely to feel heard. Knowing how to manage health and what to expect is a key component of their interactions with the provider/team. It seems that there is always some division between health care and social care which is why they sit at opposite sides of the jagged line. We define health care as support for medical concerns, symptoms and activities of daily living (bathing, mobility, etc.). Social care is defined as instrumental activities of daily living (e.g., housekeeping, meal planning, paying bills, etc.), respite care, socialization, as well as social determinants (including housing and food security). Balancing safety and independence is a key goal for caregivers and providers but tend to trade-off. For example, caregivers and providers tend to prioritize safety while patients prioritize independence. Also, as caregiver’s strive to keep their loved ones safe, they too, may feel a loss of independence.

## Findings in relation to previous work

The relational attribute in our study (feeling heard, appreciated and comfortable) coincide with key tenets of person centered care [8,9,10] also described in the early works of Balint [40] and Rogers [41]. It also reflects more meaningful engagement as described in the patient engagement continuum by Carman et al. [11], as it moves beyond consultation to involvement and partnership. Mead and Bower [9] conducted a review of the empirical and conceptual literature of patient centred care, with a focus on the patient-doctor relationship. They identified 5 dimensions: biopsychosocial perspective, ‘patient as person’, sharing power and responsibility, therapeutic alliance and doctor as person. Similarly, in a recent narrative review, Santana et al. [14] synthesized the patient centred care literature by Donabedian’s structure-process-outcome framework. Structural variables included factors such as supporting a workforce committed to patient centred care and structures to support health information technology across sectors; process variables included respectful and compassionate care and engaging patients and families; and outcome variables included access to care and patient reported outcomes. These findings align with our work while drawing attention to the structural components required to enable patient and caregiver centred attributes to be realized.

Our findings are also supported by a qualitative study conducted by Bayliss et al. [42] of 26 community dwelling older adults with multimorbidity who wanted easy access to their care providers, clear communication of care plans that were tailored to their needs, support from a single coordinator, and providers who listened to them.

Finally, our work coincides with and extends an emerging body of literature on goals of care. Goals of care, often elicited from questions directed to patients such as “What matters to you?” often reveal things that are social in nature, as opposed to medical. Literature on goals of care reveals that people may rather face death than lose their independence [43]. As pointed out by Bernsten et al. health goals and social goals are intricately connected as health issues may get in the way of achieving life oriented or social goals [44]. We saw this in our study as patients and caregivers both voluntarily and involuntarily adjusted their routines and hobbies of interest due to their health issues. In a recent study, Vermunt et al. [45] outlined a 3 goal model for patients: disease or symptom level goals; functional goals as well as fundamental goals (patient priorities and values). Our paper extends this work by exploring what these fundamental goals look like and how they might be operationalized in Community Based Primary HealthCare for older adults with complex needs.

The activities and attributes of outcomes that matter to people (summarized in Table 2) can support the implementation of programs that are designed with the user and their families in mind [11]. Our study also demonstrates how these outcomes overlap and trade-off with one another.

## Limitations

This paper provides an analysis of high level attributes that were identified by both patients and caregivers across multiple jurisdictions within two countries providing insight into things that matter to people, generally. Our paper does not provide a comparative analysis of attributes, by key sub-groups such as patients, caregivers, or geographical factors (jurisdictions) or individual characteristics (ethnicities, language groups, etc.) which would help to answer important implementation questions including, what works for whom and in what conditions? Future work of the team will consider the perspectives of providers, organization leads and decision makers; required to inform the implementation of person centred CBPHC.

## Conclusions

Many key attributes of good care extend beyond the management of disease. The importance of comfortable and reliable relationships, being independent, having access to social care resources and the trade-offs that patients and caregivers make as their needs change need to be considered as health care systems seek to better coordinate and integrate care for vulnerable populations and their families. While our findings shed light on activities that characterize these attributes, further research on implementation barriers and facilitators is required. The analysis presented in this paper is the fundamental first step in understanding core attributes that should be considered in the design and delivery of care from the perspectives of people with complex care needs and their caregivers.
